# Changes in levels of angiotensin II and its receptors in a model of inverted stress-induced cardiomyopathy

**DOI:** 10.1186/s40001-014-0054-8

**Published:** 2014-10-09

**Authors:** Yin-yan Xi, Bei Liu, Li-xia Yang, Chen-wei Kuang, Rui-wei Guo

**Affiliations:** Department of Cardiology, Kunming General Hospital of Chengdu Military Command, Daguan Road, No212, Yunnan, 650032 China; Department of Obstetrics and Gynecology, Kunming General Hospital of Chengdu Military Command, Daguan Road, No212, Yunnan, 650032 China

**Keywords:** Stress-induced cardiomyopathy, Angiotensin II, Angiotensin II receptors, Renin-angiotensin system, Angiotensin (1-7)

## Abstract

**Background:**

Stress-induced cardiomyopathy (SIC) has gained increasing attention worldwide and is characterized by extensive ventricular akinesis, Beta-blockers and angiotensin-converting enzyme inhibitors (ACEIs) are the main treatments for SIC patients. The pharmacological mechanism of action of beta-blockers results in the inhibition of the biological effects of catecholamines. However, the mechanism of action of ACEIs in the treatment of cardiomyopathy is not known. Our aim is to assess changes in levels of angiotensin II, angiotensin-II receptors and ACE responses to SIC.

**Methods:**

A model of inverted SIC was established in rabbits by vagal electrical stimulation. The serum concentration of angiotensin II and angiotensin (1-7) was detected by enzyme-linked immunosorbent assay. Expression of angiotensin-II receptors was measured by Western blotting and real-time reverse transcription-polymerase chain reaction (RT-PCR), with localization detected by immunofluorescent staining. ACE-II expression in the myocardium was measured by Western blotting.

**Results:**

From one day after vagal stimulation, concentrations of angiotensin II were significantly higher in the experimental group than those in the control group (P <0.05). Stress induced a time-dependent decrease in angiotensin subtype-1 (AT1) expression and a time-dependent increase in AT2 expression only in the apical portion of the myocardium. From three days after vagal stimulation, angiotensin (1-7) levels were significantly lower in the experimental group compared with the control group (P <0.05). Expression of the ACE-II protein was significantly downregulated in the experimental group compared with the control group from three days after vagal stimulation (P <0.05).

**Conclusions:**

Expression of angiotensin II, its receptors, ACE-II and angiotensin (1-7) was altered in response to SIC. The renin-angiotensin system could represent a therapeutic target in the prevention of SIC.

**Electronic supplementary material:**

The online version of this article (doi:10.1186/s40001-014-0054-8) contains supplementary material, which is available to authorized users.

## Background

Stress-induced cardiomyopathy (SIC; also named Takotsubo cardiomyopathy) was first described in Japan in 1991 [1]. Stress factors can be sudden psychological, physical or emotional. SIC has gained increasing attention worldwide and is a well-recognized cause of acute heart failure, lethal arrhythmias and ventricular rupture [2]. SIC is characterized by extensive ventricular akinesis involving apical segments with preserved function in basal segments without significant lesions in coronary arteries.

Recently, evidence has shown that serum levels of catecholamines in SIC patients one to two days after presentation are higher than in patients with myocardial infarction with pulmonary edema, and that epinephrine levels return to those seen in those with myocardial infarction only after seven to nine days [3]. Experimental studies reported the catecholamine intoxication is the main etiology of SIC [4, 5], but the mechanism of action is not clear.

SIC patients have an excellent prognosis as well as absent or minimal residual cardiac impairment, and left ventricular (LV) function recovers within two to four weeks of presentation [6]. At present, beta-blockers and angiotensin-converting enzyme inhibitors (ACEIs) are the main treatments for SIC patients [3, 7, 8]. The pharmacological mechanism of action of beta-blockers results in the inhibition of the biological effects of catecholamines [9]. However, the mechanism of action of ACEIs in the treatment of cardiomyopathy is not known. The target of ACEIs is angiotensin-converting enzyme 1 (the main function of which is to convert angiotensinogen to angiotensin II). Angiotensin II contains peptides that are involved in the renin-angiotensin system (RAS) and which regulate cellular and physiological responses in the cardiovascular system. Angiotensin II is also implicated in the pathophysiological processes related to myocardial remodeling [10, 11]. Angiotensin II activates several nuclear transcription factors (such as signal transducer and activator of transcription (STAT) and cyclic adenosine monophosphate (cAMP) response element-binding (CREB) protein) by promoting fibroblast proliferation that leads to myocardial remodeling [12,13].

Angiotensin II signals through seven transmembrane-spanning G protein-coupled receptors that are characterized into angiotensin subtype-1 (AT1) and angiotensin subtype-2 (AT2) based on their selective affinity for peptide and non-peptide ligand [14]. Northern blotting and reverse transcription-polymerase chain reaction (RT-PCR) analyses have been used to identify AT1 and AT2 mRNA in many cells and tissues (including the human myocardium). Pharmacological studies have revealed that the AT1 subtype mediates responses to angiotensin II caused by the release of aldosterone and epinephrine [15]. One study showed that expression of angiotensin-II receptors changes in response to stress [16]. Nevertheless, few studies have focused on the association between angiotensin II and its receptors with SIC.

In the present study, we assessed changes in angiotensin II, angiotensin-II receptors, and angiotensin-converting enzyme (ACE) responses to SIC from *in vivo* studies using a rabbit model of SIC.

## Methods

All studies conformed to the *Guide for the Care and Use of Laboratory Animals* (US National Institutes of Health, publication number 85-23, revised 1996; Additional file 1: Figure S1).

### Reagents

Anti-AT1 and anti-AT2 receptor antibodies were purchased from Santa Cruz Biotechnology (Santa Cruz, California, United States). A one-step RT-PCR kit was obtained from TaKaRa (TaKaRa, Shiga, Japan). Angiotensin II, angiotensin (1-7) and ACE-II enzyme-linked immunoassay (ELISA) kits (all from rabbits) were obtained from Blue Gene Chemical Company (Blue Gene Chemical Company, Shanghai, China). Tween 20, Nonidet 40 (NP-40), aprotinin, leupeptin, phenylmethylsulfonyl fluoride (PMSF) and dithiothreitol (DTT) were purchased from Sigma-Aldrich (Sigma, St. Louis, United States).

### *In vivo* model of stress-induced cardiomyopathy

Experimental procedures were designed according to those of Takato et al. [17]. Female rabbits (weighing approximately 2 kg; General Hospital of Chengdu Military Command, Kunming, China) were anesthetized (100 mg/kg ketamine, 5 mg/kg xylazine(Aibixin Chemical Company, Shanghai, Chin), intramuscular injection). Electrical stimulations of 50-Hz intensity and l-ms duration with stepwise increases in voltage from 0.1 to 1.0 V were applied to the right cervical intact vagus under electrocardiographic monitoring. Stimulation was maintained for 1 minute with a pause of 2 minutes between stimulations for approximately 1 hour. The sham group did not have electrical stimulations. At 1, 3, 7 and 14 days after vagal stimulation, animals were anesthetized and hearts removed.

### Western blotting

Proteins were extracted as described [18]. 100 ug of protein were separated by sodium dodecyl sulfate-polyacrylamide gel electrophoresis (10% polyacrylamide gel (Xibao Chemical Company, Shanghai, China)). Proteins were transferred onto polyvinylidene difluoride membranes by electroblotting for 3 hours at 150 mA. Membranes were blocked in 5% non-fat milk solution in Tris-buffered saline with 0.5% Tween 20 (Sigma, St. Louis, United States). Membranes were allowed to react with primary antibodies (respectively AT1 and AT2 antibody). Detection of specific proteins was done by enhanced chemiluminescence following manufacturer instructions. Densitometric signals were quantified using Quantity One software (BioRad, Hercules, California, United States).

### RNA isolation and real-time reverse transcription-polymerase chain reaction

Total RNA was isolated with TRIzol™ reagent according to manufacturer protocols (Sigma, St. Louis, United States). Total RNA was reverse-transcribed into cDNA. The resultant cDNA was amplified by SYBR Green 1 fluorescence real-time RT-PCR. The PCR reaction was monitored directly using the Bioer FQD-66A sequence detection system(Bioer Company, Hangzhou, China).

The primers for AT1 were 5′-TTTGGGAACAGCTTGGCGGT-3′ (forward) and 5′-GCCAGCCAGCAGCCAAATAA-3′ (reverse). The primers for AT2 were 5′-AGGTTTCCAGCATTTACATC-3′ (forward) and 5′-GTCACCAGCCAACGCTATC-3′ (reverse). The primers for β-actin were 5′-AGGAAGGAGGGCTGGAACA-3′ (forward) and 5′-CCCATCTACGAGGGCTACGC-3′ (reverse).

3 ug single-stranded cDNA was amplified by PCR using 35 cycles. The PCR profile used for AT1 amplification was 30 s of denaturation at 94°C, 30 seconds of annealing at 57°C, and 1.5 minutes of extension at 72°C. The PCR profile used for AT2 amplification was 30 seconds of denaturation at 94°C, 30 seconds of annealing at 60°C, and 1.5 minutes of extension at 72°C. The profile for β-actin amplification was: 30 s of denaturation at 94°C, 30 seconds of annealing at 57°C, and 1.5 minutes of extension at 72°C. Results were expressed as the mean ± SD for relative expression levels.

### Immunofluorescence

Apical tissue was removed and used for detection of AT1 and AT2 by immunofluorescence. For staining, sections were fixed in acetone for 10 minutes, air dried, and rehydrated with phosphate-buffered saline (PBS) before incubation in serum-free Protein Block (Dako, Glostrup, Denmark) for 30 minutes. Sections were stained with antibody diluted in 1% blocking reagent/0.3% Triton X-100 in PBS overnight before being washed in Tris-NaCl-Tween-Buffer (TNT) wash buffer (Tris-HCl, pH 7.5, 0.15 mol/L NaCl, and 0.05% Tween 20 (Sigma, St. Louis, United States). Sections incubated with an isotype-matched control antibody were used as the negative control. Subsequently, sections were incubated with fluorescein isothiocyanate-goat anti-mouse IgG(Zhongshan, Beijing, China) for 30 minutes before washing. Nuclei were stained with 4′,6-Diamidino-2-phenylindole dihydrochloride. Images were obtained using a Confocal Laser Scanning Microscope (TCS SP5; Leica, Wetzlar, Germany).

### ELISA

The concentration of angiotensin II and angiotensin (1-7) in serum samples was detected by ELISA according to specifications in the kit (Sigma, St. Louis, United States).

### Statistical analyses

Results are the mean ± SEM. SPSS version 13.0 (SPSS, Chicago, Illinois, United States) was used for statistical analyses. The Student *t*-test was employed to compare two groups. Analysis of variance (ANOVA) was used with the Tukey multiple comparison test for multiple groups. A *P* value of <0.05 was considered significant.

## Results

### Change in plasma levels of angiotensin II in response to stress

**Figure 1:**
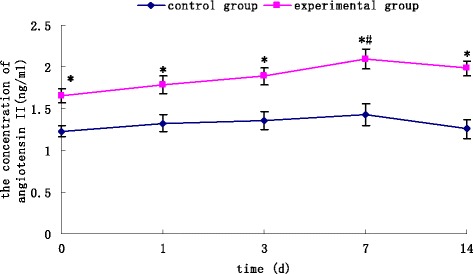
**Changes in plasma levels of angiotensin II in response to stress.** The plasma concentration of angiotensin II was measured by ELISA. Concentrations of angiotensin II were significantly higher in the experimental group than those in the control group from 0 days after vagal stimulation. Seven days after vagal stimulation, the concentration of angiotensin II reached its peak, and was significantly higher than at 0 days after vagal stimulation. Results are the mean ± SEM of six experiments. ^*^P <0.05 *versus* control groups ^#^P < 0.05 *versus* at day 0 after vagal stimulation.

At 0, 1, 3, 7 and 14 days after vagal stimulation, the plasma of rabbits in control and experimental groups was extracted, and the concentration of angiotensin II measured by ELISA. From 1 day after vagal stimulation, the concentration of angiotensin II increased gradually in the experimental group. At seven days, the concentration of angiotensin II reached its peak (1.353 ± 0.003 ng/mL), and was significantly higher than that 0 days after vagal stimulation (P <0.05). Meanwhile, the concentrations of angiotensin II were significantly higher in experimental group than those in the control group from one day after vagal stimulation (P <0.05). Detailed data was listed in Figure 1.

### Expression of AT1 and AT2 mRNA and protein in a model of acute cardiomyopathy

**Figure 2:**
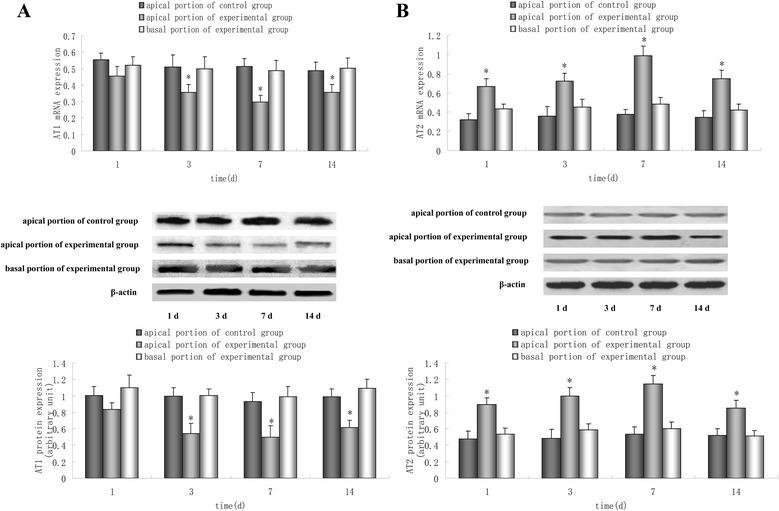
**Real-time RT-PCR and Western blotting for expression of AT1 (A) and AT2 (B) mRNA and protein, respectively.** Data are representative of three different experiments. Compared with that in the apical portion of the control group and basal portion of the experimental group, expression of AT1 mRNA and protein was downregulated significantly in the apical portion of the experimental group from three days after vagal stimulation, but expression of AT2 mRNA and protein in the apical portion was upregulated from one day after vagal stimulation. Results are the mean ± SEM of three experiments. ^*^P <0.05 *versus* apical portion of the control group and basal portion of the experimental group.

We next examined the effect of stress on angiotensin receptor mRNA and protein expression. From one day after vagal stimulation, stress induced a time-dependent decrease in the expression of AT1 mRNA and protein in the apical portion. Compared to that in basal portion, the expression of AT1 mRNA and protein significantly downregulated in the apical portion from three days after vagal stimulation (P <0.05). Expression of apical AT1 mRNA and protein in the experimental group was significantly lower than that in the control group (P <0.05) (Figure 2A). Conversely, expression of AT2 mRNA and protein in the apical portion was increased by stress from one day after vagal stimulation. Compared with that in the basal portion of the experimental group and the apical portion of the control group, expression of AT2 mRNA and protein was upregulated significantly in the apical portion of the experimental group (P <0.05) (Figure 2B).

### Localization of AT1 and AT2 in basal and apical portions upon response to stress

**Figure 3:**
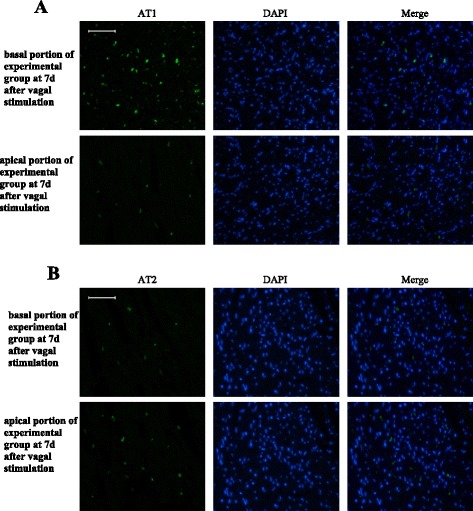
**Immunofluorescent staining to determine the localization of AT1 and AT2 in basal and apical portions of the experimental group seven days after vagal stimulation.** **A**: Immunofluorescent staining of angiotensin subtype-1 (AT1) (green) and nucleus (blue) in the myocardium. **B**: Immunofluorescent staining of angiotensin angiotensin subtype-2 (AT2) (green) and nucleus (blue) in the myocardium. Results are from a single experiment but representative of three separate experiments. All bars represent 50 μm.

Immunofluorescent staining was undertaken to determine the localization of AT1 and AT2 in basal and apical portions of the experimental group seven days after vagal stimulation. Clear immunoreactivity for AT1 and AT2 was detected in scattered cells of the myocardium in basal and apical portions. In addition, compared with those in the myocardium of the basal portion, an increased number of AT1-positive cells and a decreased number of AT2-positive cells was observed in the myocardium of the apical portion (Figure 3).

### Change in plasma levels of angiotensin (1-7) and ACE-II protein in a model of acute cardiomyopathy

**Figure 4:**
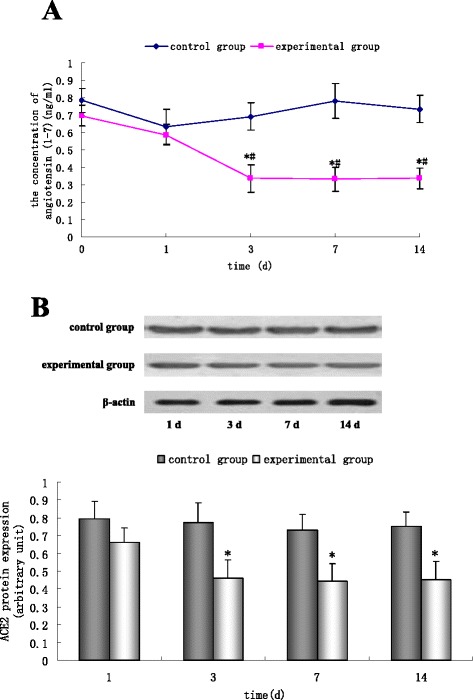
**Change in plasma levels of angiotensin (1-7) and ACE-II protein iafter vagal stimulation.** **A**, Angiotensin (1-7) levels were measured by ELISA 0, 1, 3, 7 and 14 days after vagal stimulation. **A**, Angiotensin (1-7) levels were significantly lower in the experimental group compared with the control group at 3, 7 and 14 days. Angiotensin (1-7) levels were significantly lower at 3, 7 and 14 days compared with 0 days. Results are the mean ± SEM of six experiments. ^*^P <0.05 *versus* control groups, ^#^P <0.05 *versus* at day 0 after vagal stimulation. **B**, ACE-II protein in the apical tissue of the control group and experimental group was measured by Western blotting. From three days after vagal stimulation, expression of ACE-II protein was significantly lower in the experimental group than in the control group. Results are the mean ± SEM of three experiments. ^*^P <0.05 *versus* the control group.

At 0, 1, 3, 7 and 14 days after vagal stimulation, the plasma of rabbits in control and experimental groups was extracted, and the concentration of angiotensin (1-7) measured by ELISA. From three days after vagal stimulation, angiotensin (1-7) levels were significantly lower in the experimental group compared with the control group (P <0.05). When compared with 0 days after vagal stimulation, the concentration of angiotensin (1-7) was significantly lower at 3, 7 and 14 days (P <0.05) (Figure 4A). Apical tissue of the control group and experimental group was collected, and levels of ACE-II protein measured by Western blotting. Compared with that in the control group, expression of ACE-II protein was downregulated significantly in the experimental group from three days after vagal stimulation (P <0.05) (Figure 4B).

## Discussion

The RAS has been shown (clinically and experimentally) to be associated with the progression and outcome of cardiovascular diseases such as atherosclerosis, acute coronary syndrome, cardiac hypertrophy and heart failure [19, 20]. Angiotensin II has been implicated in the pathophysiological processes related to myocardial remodeling [11, 21]. The link that connects SIC and RAS is not known.

In the context of former studies on cardiomyopathy and the rennin angiotensin system, our study elicited three main findings. Firstly, the concentration of angiotensin II increased gradually in the rabbit model of inverted SIC by vagal stimulation. Secondly, expression of apical AT1 mRNA and protein in the experimental group was decreased significantly, but expression of AT2 mRNA and protein was upregulated significantly. Finally, plasma levels of angiotensin (1-7) and ACE-II expression were decreased in the rabbit model of inverted SIC.

Recently, epinephrine-induced cardiotoxicity has been postulated to be involved in the pathogenesis of SIC [5, 22]. It is well established that angiotensin II can stimulate epinephrine release from the adrenal glands [15, 23]. The present study is the first to show that the plasma concentration of angiotensin II increases gradually in SIC. These data strongly suggest that angiotensin II and its signaling pathway may be involved in the pathogenesis of SIC. Nevertheless, the underlying mechanism of action merits further study.

Premer et al. revealed that the AT1 subtype mediates the release of aldosterone and epinephrine as well as pressor, dipsogenic and sodium-appetite responses to angiotensin II in the brain [24]. We examined the effect of stress on the expression of mRNA and proteins from angiotensin receptors. The results showed that stress induced a time-dependent decrease in the expression of AT1 mRNA and protein in only the apical portion. Conversely, expression of AT2 mRNA and protein in the apical portion was increased by stress. Immunofluorescent staining showed AT1 and AT2 in scattered parts of the myocardium. Izumi et al. also reported that expression of AT1 mRNA was significantly reduced, and that of AT2 remarkably increased, in apical portions [16]. Several lines of evidences suggest that catecholamine concentrations are increased by stress [25, 26]. Downregulation of expression of AT1 and upregulation of expression of AT2 in the apical portion of the myocardium may be induced by excess epinephrine [16]. Data have shown expression of AT1 and AT2 in the left ventricle to be decreased in human heart failure, which is different to the effect of stress on expression of AT1 and AT2 [27]. AT2 mediates cardioprotection, so upregulation of expression of AT2 in the apical portion of the myocardium could (at least in part) influence the prognosis of patients with SIC.

ACE-II is a homolog of ACE compounds, and has been found to be present in the heart, kidney and other tissues [28]. The bioactivity of ACE-II helps to catalyze angiotensin II to angiotensin (1-7). Angiotensin (1-7) is a cardiovascular-protective peptide with vasodilatory and antiproliferative effects. ACE-II can increase the degradation of angiotensin II, thereby reducing the adverse effects of angiotensin II [29,30]. Our study is the first to show that angiotensin (1-7) levels were significantly lower in response to SIC. Expression of ACE-II proteins was also significantly downregulated in the apical myocardium in SIC, and is part of the cause of stress-induced heart failure.

## Conclusions

Expression of angiotensin II, its receptors, ACE-II and angiotensin (1-7) was altered in response to SIC. The renin-angiotensin system could represent a therapeutic target in the prevention of SIC.

## Additional file

Additional file 1: Figure S1. A model of stress-induced cardiomyopathy was established in rabbits by vagal electrical stimulation according to the method of Takato et al. [17]. Female rabbits (weighing approximately 2 kg; General Hospital of Chengdu Military Command, Kunming, China) were anesthetized (100 mg/kg ketamine, 5 mg/kg xylazine, intramuscular injection). The right cervical vagus was exposed (A). Electrical stimulations of 50-Hz intensity and l-ms duration with stepwise voltage increases from 0.1 to 1.0 V were applied to the right cervical vagus under electrocardiographic monitoring. Stimulation was maintained for 1 minute with a pause of 2 minutes between each stimulation for approximately 1 hour (B).

## Abbreviations

ACEIs: Angiotensin-converting enzyme inhibitors
AT1: Angiotensin subtype-1
AT2: Angiotensin subtype-2
cAMP: Cyclic adenosine monophosphate
DTT: Dithiothreitol
ELISA: Enzyme-linked immunosorbent assay
PMSF: Phenylmethylsulfonyl fluoride
RT-PCR: Real-time reverse transcription-polymerase chain reaction
RAS: Renin-angiotensin system
CREB: Response element-binding
STAT: Signal transducer and activator of transcription
SIC: Stress-induced cardiomyopathy
TNT: Tris-NaCl-Tween-Buffer
