# Supplementary material for: Changes in levels of angiotensin II and its receptors in a model of inverted stress-induced cardiomyopathy
Source: Eur J Med Res. 2014 Oct 9;19(1):54. doi: 10.1186/s40001-014-0054-8 (PMC4240840; doi:10.1186/s40001-014-0054-8)
Supplement: Additional file 1: Figure S1. — A model of stress-induced cardiomyopathy was established in rabbits by vagal electrical stimulation according to the method of Takato et al. [17]. Female rabbits (weighing approximately 2 kg; General Hospital of Chengdu Military Command, Kunming, China) were anesthetized (100 mg/kg ketamine, 5 mg/kg xylazine, intramuscular injection). The right cervical vagus was exposed (A). Electrical stimulations of 50-Hz intensity and l-ms duration with stepwise voltage increases from 0.1 to 1.0 V were applied to the right cervical vagus under electrocardiographic monitoring. Stimulation was maintained for 1 minute with a pause of 2 minutes between each stimulation for approximately 1 hour (B). [file 40001_2014_54_MOESM1_ESM.doc]

**Additional file 1**

**Supplementary Figure Legends:**

A model of stress-induced cardiomyopathy was established in rabbits by vagal electrical stimulation according to the method of Takato et al. Female rabbits (≈2 kg; General Hospital of Chengdu Military Command, Kunming, China) were anesthetized (100 mg/kg ketamine, 5 mg/kg xylazine, i.m.). The right cervical vagus was exposed **(A)**. Electrical stimulations of 50-Hz intensity and l-ms duration with stepwise voltage increases from 0.1 V to 1.0 V were applied to the right cervical vagus under electrocardiographic monitoring. Stimulation was maintained for 1 min with a pause of 2 min between each stimulation for ≈1 h **(B)**.

**Supplemental Figure**


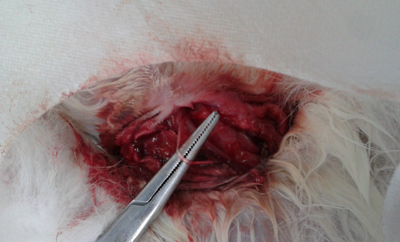


**A**


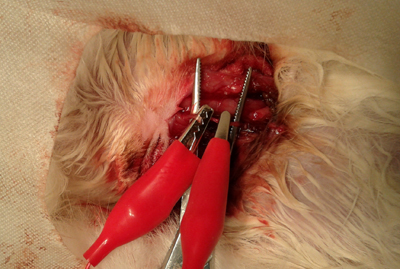


**B**
